# Water-induced MAPbBr_3_@PbBr(OH) with enhanced luminescence and stability

**DOI:** 10.1038/s41377-020-0283-2

**Published:** 2020-03-17

**Authors:** Kai-Kai Liu, Qian Liu, Dong-Wen Yang, Ya-Chuan Liang, Lai-Zhi Sui, Jian-Yong Wei, Guo-Wei Xue, Wen-Bo Zhao, Xue-Ying Wu, Lin Dong, Chong-Xin Shan

**Affiliations:** 1 Henan Key Laboratory of Diamond Optoelectronic Materials and Devices, Key Laboratory of Material Physics, Ministry of Education, School of Physics and Microelectronics, Zhengzhou University, Zhengzhou, 450052 China; 2 State Key Laboratory of Molecular Reaction Dynamics, Dalian Institute of Chemical Physics, Chinese Academy of Sciences, 457 Zhongshan Road, Dalian, 116023 China

**Keywords:** Optical materials and structures, Nanoparticles

## Abstract

Poor stability has long been one of the key issues that hinder the practical applications of lead-based halide perovskites. In this paper, the photoluminescence (PL) quantum yield (QY) of bromide-based perovskites can be increased from 2.5% to 71.54% by introducing water, and the PL QY of a sample in aqueous solution decreases minimally over 1 year. The enhanced stability and PL QY can be attributed to the water-induced methylamino lead bromide perovskite (MAPbBr_3_)@PbBr(OH). We note that this strategy is universal to MAPbBr_3_, formamidine lead bromide perovskite (FAPbBr_3_), inorganic lead bromide perovskite (CsPbBr_3_), etc. Light-emitting devices (LEDs) are fabricated by using the as-prepared perovskite as phosphors on a 365 nm UV chip. The luminance intensity of the LED is 9549 cd/m^2^ when the driven current is 200 mA, and blemishes on the surface of glass are clearly observed under the illumination of the LEDs. This work provides a new strategy for highly stable and efficient perovskites.

## Perovskite LEDs: extending lifetimes with a splash of water

Inexpensive crystals useful for applications including display devices can be manufactured with exceptional environmental stability by following a new synthetic technique. Lead halide perovskites are materials that emit light with a range of colors, but also suffer from poor moisture stability. Kai-Kai Liu, Lin Dong, Chong-Xin Shan from China’s Zhengzhou University and colleagues now report that adding a dose of water to lead halide perovskites enables them to emit bright fluorescent light, even when submerged underwater for more than a year. The team’s experiments revealed that controlled hydration of the perovskite’s outer surface helped remove defects that decrease light output, as well as protected against further incursions by air and water molecules. Successful fabrication of the hydrated crystals as phosphors within light-emitting devices (LEDs) indicates their potential for industrial purposes.

## Introduction

In recent years, lead halide perovskites (LHPs) APbX_3_ (A = CH_3_NH_3_^+^/CH(NH_2_)_2_^+^/Cs^+^, X = Cl^−^/Br^−^/I^−^) have emerged as promising materials for photovoltaics and light-emitting diodes due to their attractive optical and electrical properties, such as high photoluminescence (PL) quantum yield (QY), narrow emission spectrum, tuneable emission wavelength, high absorption coefficient, and long carrier diffusion length^1–11^. Profound developments have been witnessed in the fields of solar cells^12–15^, solid-state light-emitting diodes^11,16–20^, photodetectors^21–23^, and lasers^7,24,25^. However, the poor stability of LHPs, especially in water and polar solvents, remains a crucial issue that hampers their applications.

The origin of the instability of LHPs is generally attributed to their low formation energy, which makes these materials vulnerable to light, heat, oxygen, and moisture, especially when water is involved^26–29^. In addition, the ions of LHPs with discrete components are exchanged with each other quickly, which results in severe shifts in the emissions. Strategies for improving the stability of LHPs can be classified into three types: compositional engineering, surface engineering, and matrix encapsulation^30^. The all-inorganic lead perovskite (CsPbBr_3_), where methylamino (MA) ions are replaced with Cs ions, shows much higher stability, including thermal and environmental stability, than organic–inorganic perovskite^31^. Although the thermal and environmental stability of this perovskite has been improved through compositional engineering, poor moisture stability is still a serious problem. The stability of LHPs improves greatly when they are covered with long-chain surface agents, which has been widely demonstrated by researchers. However, LHPs usually lose surface ligands and then reunite and lose their colloidal stability during purification^32^. Matrix encapsulation has been applied to effectively enhance the stability and PL efficacy of perovskite. For example, Jia et al. prepared CsPbX_3_/Cs_4_PbX_6_ core/shell perovskite nanocrystals by applying a seeded growth approach, which provided improved PL QY. Wang et al. produced a Rb_4_PbBr_6_ shell to protect CsPbX_3_ through the rubidium oleate posttreatment method. Similarly, Tang et al. reported a kind of CsPbBr_3_/CdS core/shell structure using a hot-injection method. Embedding CsPbBr_3_ into SiO_2_ or the pores of mesoporous silica can also improve the stability of the perovskite^33–37^. Although most inorganic matrices are dense and thermally stable, it is difficult to controllably form an inorganic protective layer covering perovskite quantum dots (QDs) due to the high synthetic temperature and relatively complicated synthesis conditions. Furthermore, the introduction of expensive or toxic elements further hinders the applications of perovskites. Moreover, the phase separation between LHPs and protection media still remains, resulting in size variation, low loading, broad spectra, and low PL QY. Some researchers have demonstrated the positive role of water in the synthesis of LHPs^38–40^. For example, Andrey et al. synthesized stable CsPbBr_3_ nanocrystals by introducing a suitable amount of water into the reaction mixture^41^. It is important to note that when a large amount of water was introduced in this system, the perovskite still decomposed. Atanu and Kwang demonstrated the aqueous synthesis of various hybrids and all-inorganic halide perovskites in acidic and basic media^42^. The perovskites were stable in water for more than 6 months, but the PL QY of the as-prepared methylamino lead bromide perovskites (MAPbBr_3_) was only 11.7%. These observations indicate that there exists a solubility equilibrium between the crystallization of perovskite and its saturated ionic components in water. Thus, the luminescence and stability of LHPs may be enhanced by water by choosing appropriate pH, metal halide salts, and different organic components.

In this work, we show that the PL QY and stability of LHPs can be greatly enhanced by adding water, and the PL QY of the LHPs can be increased from 2.50% to 71.54%, while that of a sample in aqueous solution decreases minimally after 1 year. This strategy is universal to MAPbBr_3_, formamidine lead bromide perovskites (FAPbBr_3_), and CsPbBr_3_. UV-pumped LEDs have been fabricated by using the prepared perovskites as phosphors, and blemishes including scratches, dust, and fingerprints on the surface of glass can be observed clearly under the illumination of the LEDs, indicating that the LEDs are suitable for manual defect detection.

## Results

### Structural and morphology characterization

**Fig. 1:**
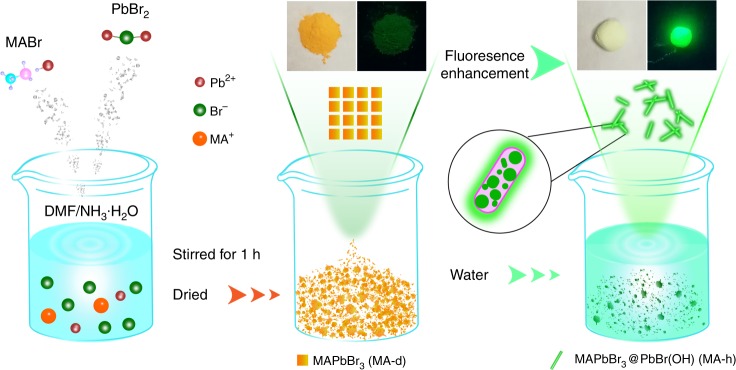
Schematic diagram of the synthesis process for water-induced MAPbBr_3_@PbBr(OH)

The schematic synthesis process for the MAPbBr_3_@PbBr(OH) is illustrated in Fig. 1. The pH value of *N*,*N*-dimethylformamide (DMF) was preadjusted to 9.0 through dropwise addition of ammonium hydroxide; a mixture of PbBr_2_ and MABr with a mole ratio of 1.05:1 was dissolved in DMF solvent under continuous stirring until a white precipitate was formed. Subsequently, the precipitate was placed into an oven at 70 °C to obtain dried MAPbBr_3_ perovskite, as shown in the middle of Fig. 1. The dried MAPbBr_3_ perovskite is named MA-d for convenience, and the yellow MA-d exhibits very weak fluorescence under UV illumination. After addition of water, bright green fluorescence appears. Detailed characterizations indicate that MA-d changes to rod-shaped PbBr(OH), and MAPbBr_3_ QDs are embedded in situ into the PbBr(OH) microrods to form MAPbBr_3_@PbBr(OH). MAPbBr_3_@PbBr(OH) is named MA-h for convenience. Based on the first-principles calculations, the bandgap of PbBr(OH) is ~3.1 eV, which is close to the bandgap of the material (3.44 eV). The valence band maximum originates from the Br and O orbitals, and the conduction band (CB) minimum is dominated by the Pb orbitals, as shown in Supplementary Fig. 1. The decomposition enthalpies of MAPbBr_3_ and PbBr(OH) are 0.38 and 16.15 eV, respectively, (Supplementary Fig. 2, Supplementary Table 1), indicating that PbBr(OH) has higher thermodynamic stability than MAPbBr_3_, which can prevent the decomposition of internal MAPbBr_3_ QDs. Different from that apt to degrade and lose fluorescence in the presence of moisture^43^, the MA-h synthesized in this work can maintain its bright fluorescence for a year in water.

**Fig. 2:**
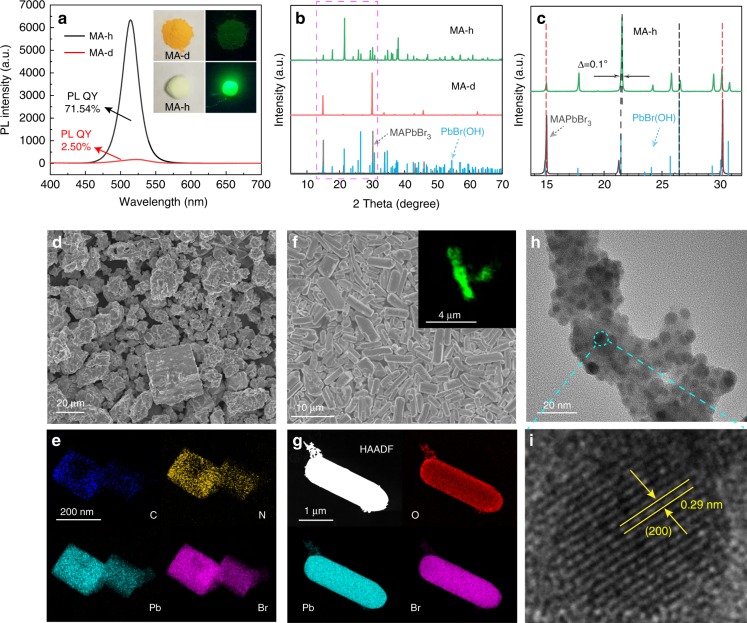
**a** PL spectra of MA-d (PL QY: 2.50%) and MA-h (PL QY: 71.54%), and the inset shows the corresponding optical images of the two samples under indoor lighting and UV illumination. **b** XRD patterns of MA-d and MA-h. **c** Magnification of regions marked in **b**. **d** SEM image of MA-d. **e** Elemental mapping of MA-d. **f** SEM image of MA-h, and the inset shows a laser confocal fluorescence microscopy image. **g** Elemental mapping of MA-h. **h** TEM image of the crushed MA-h. **i** HRTEM image of the QDs in MA-h

The MA-d powders are yellow in color and show negligible green fluorescence under UV illumination. The MA-h powders are a greenish color and show bright green fluorescence under UV illumination, and the corresponding picture is shown in the inset of Fig. 2a. The fluorescence spectra of MA-d and MA-h are shown in Fig. 2a, and the emission intensity of MA-h increases 35-fold compared with that of MA-d. To compare the PL peaks of MA-d and MA-h, their PL intensities were normalized, as shown in Supplementary Fig. 3. The PL peak of MA-d is located at 523 nm, while that of MA-h is located at 514 nm, indicating that the size of MA-d decreases after adding water. In addition, the full-width at half-maximum (FWHM) of MA-d is 51 nm, and the FWHM of MA-h is only 26 nm, indicating the smaller and uniform size distribution of MA-h. The crystal structures of MA-d and MA-h were characterized by X-ray diffraction (XRD), as shown in Fig. 2b. All the diffraction peaks of MA-d are from the cubic perovskite structure of MAPbBr_3_, while the spectrum of MA-h contains many extra peaks in addition to those of MAPbBr_3_, and the extra peaks can be assigned to PbBr(OH) (JCPDS No. 89–2492), indicating the formation of PbBr(OH). The enlarged XRD pattern (Fig. 2c) reveals that the diffraction peaks slightly shift to higher angles by ~0.1° compared with the standard data of PbBr(OH), which might be a result of the lattice mismatch between PbBr(OH) and MAPbBr_3_. To further investigate the structural evolution of the samples before and after adding water, scanning electron microscopy (SEM) images and transmission electron microscopy (TEM) images were taken. Before adding water, MA-d is a typical cubic structure (Fig. 2d), which matches well with its XRD results. To further explore the microscopic morphology of the samples, the MA-d powders were broken by an ultrasonic cell crusher in toluene. In the TEM image of the crushed MA-d (Supplementary Fig. 4), monodisperse and uniform QDs (10 nm in size) with cubic shapes can be observed, and the QDs tend to agglomerate on the TEM grids, as previously reported^44^. The inset of Supplementary Fig. 4 highlights that MA-d possesses a well-defined crystalline structure with a characteristic lattice distance of 0.58 nm, corresponding to the d-spacing of the (100) crystal planes of MAPbBr_3_. C, N, Pb, and Br can be observed from the elemental mapping of MA-d (Fig. 2e); the elements of C and N come from methylamine, while the elements of Pb and Br stem from the PbBr_6_ octahedron. MA-h exhibits uniform rod morphology with an average diameter of 1.5 μm and length of 4 μm, as shown in Fig. 2f and Supplementary Fig. 5. The inset of Fig. 2f shows the corresponding laser confocal fluorescence microscope image, and uniform green fluorescence can be observed along the rod structure. Elemental mapping of MA-h is shown in Fig. 2g, and the elements O, Pb, and Br are uniformly distributed along the rod structure, which indicates the formation of PbBr(OH). To explore the inner structure of the rod, the MA-h powders were broken in a cell crusher. Some spherical QDs with an outer shell can be observed (Fig. 2h), and the lattice spacing of the QDs is 0.29 nm, corresponding to the d-spacing of the (200) crystal planes of MAPbBr_3_ (Fig. 2i). The above results confirm that the MAPbBr_3_ QDs were coated by PbBr(OH). Thus, one can conclude that the bulk cubic shape of MAPbBr_3_ changes to a rod-like shape through the addition of water, and the MAPbBr_3_ QDs are embedded into PbBr(OH). In addition, X-ray photoelectron spectroscopy (XPS) spectra were collected to detect the surface chemistry of MA-d and MA-h, as shown in Supplementary Fig. 6. The Br/Pb atomic ratios calculated from the XPS spectra are summarized in Supplementary Table 2. From the table, one can see that the Br/Pb atomic ratios of MA-d and MA-h are 3.06 and 1.11, respectively, confirming that these materials are MAPbBr_3_ and PbBr(OH).

## Discussion

### Photophysical property

**Fig. 3:**
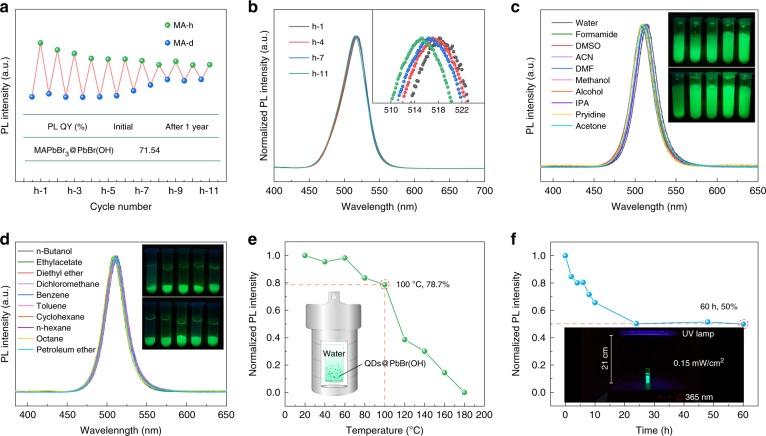
**a** The PL intensity of the sample during cycling. **b** The corresponding PL spectra of MA-h, and the inset is the magnified spectra. **c**, **d** The PL spectra and images of the MA-h powders immersed in different solvents. **e** The PL intensity of the sample in the presence of water at different temperatures, and the inset is the schematic diagram of the reaction. **f** The PL intensity of the sample in the presence of water under UV irradiation for different hours, and the inset shows the schematic diagram of the test

To evaluate the stability of the as-prepared perovskites, a repetitive hydrate–dehydrate cycle test was carried out, and the change in the PL intensity was monitored, as shown in Fig. 3a. The PL intensity of the MA-d perovskite increases instantly with the addition of water, accompanied by a color change from yellow to greenish. The perovskite gradually turns back to a yellow color after drying at 60 °C. As the hydrating–dehydrating cycle repeats, the PL intensity of MA-h decreases, while the color of the sample gradually stabilizes at greenish under ambient conditions. Additionally, the PL intensity of MA-d increases after several hydrating–dehydrating cycles, with its yellow color changing to a greenish color. The corresponding evolution of the PL spectra and the color of both samples are shown in Supplementary Figs. 7 and 8. In the first round, the PL QY of MA-d is only 2.5% and increases to 71.54% after the addition of water. The sample is held in water for 1 year, and the PL QY of the MA-h is still at 89.9% of its initial value, i.e., 64.28%, which is one of the most stable fluorescence perovskite powders ever reported to the best of our knowledge. A slight hypsochromic shift of PL is observed during the hydrating–dehydrating cycles, as evidenced by the normalized PL spectra shown in Fig. 3b. The continuous hypsochromic shift in the PL spectra might be a consequence of the decrease in the perovskite QD size during cycling. In addition, traditional LHPs are highly sensitive to polar solvents due to their inherent ionic crystal structure, and the optical properties and even structural integrity of these materials usually deteriorate in polar organic solvents^30^. However, in our work, the MA-h powders show ultrastability to many common organic solvents, such as DMSO, dimethyl formamide, ethanol, acetone, ethylacetate, and benzene. The PL spectra of the MA-h powders immersed in these organic solvents are shown in Fig. 3c, d, and the corresponding images under UV illumination are shown in the insets of Fig. 3c, d. The emission of the MA-h powders is stable in the different organic solvents, and no obvious emission peak shift is observed. In Supplementary Fig. 9, no obvious structural or morphological changes are observed in DMF or DMSO, indicating that PbBr(OH) is almost insoluble in common solvents, which is why MA-h can maintain good stability. Thermal stability is another important indicator for luminescent materials^45^, especially for LHPs. Thermo-induced particle regrowth would induce PL quenching, and the crystal structure of the perovskites would collapse directly under exceedingly high temperatures. Additionally, high temperature might accelerate the rates of oxidation and hydration, which means that the oxygen- and moisture-induced decomposition would be amplified, leading to more rapid PL quenching^30^. Fig. 3e shows the thermal stability of MA-h in the temperature range from 20 °C to 180 °C, and the corresponding PL spectra are shown in Supplementary Fig. 10. It is worth noting that the entire test process was carried out in an autoclave filled with water. It can be clearly seen that the PL intensity of MA-h decreases slowly as the temperature increases in the range of 20–100 °C, indicating good thermal stability. Upon heating above 100 °C, the PL intensity decreases sharply and eventually vanishes when the temperature reaches 180 °C. The PL decrease is due to the destruction of dense matrix PbBr(OH), and the corresponding XRD and SEM images are shown in Supplementary Fig. 11. In addition, the PL intensity of MA-h is still at 78.7% of the initial intensity upon heating from 20 °C to 100 °C. The photostability of MA-h was also investigated. Figure 3f shows the PL spectra of MA-h under continuous irradiation with a UV lamp (365 nm, 0.15 mW/cm^2^), and the corresponding PL spectra are shown in Supplementary Fig. 12. The intensity decreases sharply during the first 24 h and maintains 50% of the initial intensity after 60 h of UV illumination. Supplementary Fig. 13 shows the XRD patterns and SEM images of MA-h under UV irradiation, and no obvious structural or morphological changes can be observed. According to previous studies, a possible reason for the PL decrease under UV illumination is photoassisted ionization or photoinduced defects^46,47^. The above results indicate that MA-h has good ambient, thermal, and photostability.

### Mechanism for enhanced efficiency

**Fig. 4:**
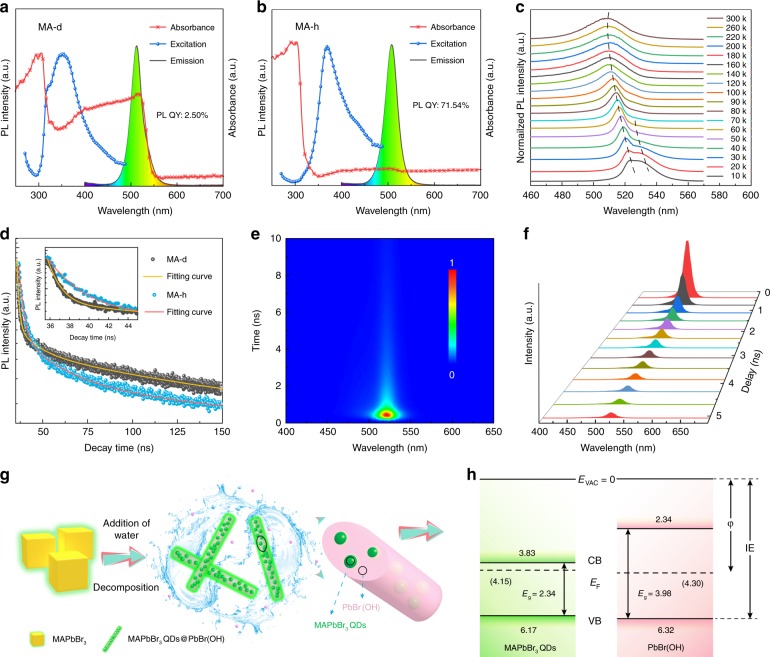
PL, PLE, and absorption spectra of **a** MA-d and **b** MA-h. **c** Temperature-dependent PL spectra. **d** PL decay curves of MA-d and MA-h, and the inset is the magnified spectra. **e** Time-resolved PL spectra of MA-h. **f** PL spectra of MA-h at different time delays. **g** Schematic illustration of the morphology evolution of the as-prepared MAPbBr_3_ perovskite. **h** Energy level diagram of PbBr(OH) and inner QDs

To further investigate the high PL QY and the luminescence mechanisms of the as-prepared perovskite in the presence of water, the steady-state PL spectra, absorption spectra, time-resolved spectra, and temperature-dependent PL spectra of the as-prepared perovskite were recorded, as shown in Fig. 4. The PL, PL excitation (PLE), and absorption spectra of the as-prepared MA-d perovskite are shown in Fig. 4a, and the samples have strong absorption at 516 nm. Figure 4b shows the PL, PLE, and absorption spectra of MA-h, from which semblable PL and excitation characteristics can be observed, while obvious differences are observed in the absorption spectra. Compared with MA-h, MA-d has a significant absorption peak at 516 nm that absorbs the emissions of MA-d. The absorption peak centered at ~310 nm with an absorption edge of 360 nm can be ascribed to the absorption of PbBr(OH). To further understand the luminescence mechanism of MA-h, temperature-dependent PL measurements were carried out with temperatures from 10 K to 300 K. In Fig. 4c, the PL peak (523 nm) at 10 K is as narrow as 6.8 nm, which originates from the emission of a typical strong excitonic recombination^31^. With the increasing temperature, the intensity of the peak centered at 530 nm (at 10 K) gradually decreases and ultimately disappears at 60 K. Additionally, a new emission peak appears in the shorter wavelength range that corresponds to free exciton emissions. By tracking the PL evolution versus temperature, we clearly reveal that the PL of MA-h at room temperature is dominated by free exciton emissions. The lifetimes of the as-prepared perovskites with and without water were recorded, as shown in Fig. 4d. Both PL decay curves of MA-d and MA-h can be fitted well by a three-exponent function, and the concrete values are summarized in Supplementary Table 3. The average lifetime of MA-h is 2.50 ns, which is longer than that of MA-d (1.08 ns). This result is supported by the time-resolved spectrum of MA-h, as shown in Fig. 4e. The longer PL lifetime and higher PL intensity of MA-h indicate a lower trap-state density^48^, which suggests that PbBr(OH) effectively passivates the defect sites in MAPbBr_3_ perovskite. In addition, oxygen molecules can increase the PL efficiency in bulk structures but have the opposite effect in QDs^49^. The instability of halide perovskite caused by ion migration is a possible issue^50^. Ion migrations are usually caused by halide vacancies, and the halide vacancies are passivated by the Br ions of PbBr(OH) in this work. Therefore, this strategy can decrease the instability caused by ion migration. Figure 4f shows the 3D plot of the PL spectra at various times, revealing an essentially time-independent spectral shape of PL emissions. In addition, the FWHM and emission peak are recorded in Supplementary Fig. 14, and the results suggest an absence of peak shifting or broadening.

To further investigate the formation mechanism of MA-h in the presence of water, we studied the morphological evolution of MA-h, and a possible mechanism was proposed. The morphological evolution of MA-h during the drying process was observed by optical microscopy and fluorescence microscopy. In Supplementary Fig. 15, the MA-h self-assembles into a cubic structure with loss of water, and then the rod structure forms again after water is added. Figure 4g shows a schematic illustration of the formation process of the as-prepared sample in the presence of water. Initially, yellow bulk perovskite with NH^4+^ and OH^−^ attached to the surface is produced. With the addition of water, the yellow bulk perovskite is destroyed and decomposes from the outside to inside, and PbBr(OH) is simultaneously formed with the aid of OH^−^ during this process. A portion of the decomposed small crystals (QDs) are wrapped into rod-shaped PbBr(OH) by oriented self-assembly. The sample exhibits improved PL QY and ultrahigh stability due to the formation of MAPbBr_3_ QDs embedded into the PbBr(OH) matrix. With the volatilization of water during the drying process, PbBr(OH) reacts with precipitated MABr (MABr is soluble in water) to form cubic perovskite. Correspondingly, the color of the sample changes from greenish to yellow, and the fluorescence intensity decreases after drying. Subsequently, each cycle (adding water and drying) produces a similar phenomenon until all the MABr is taken away by water and the perovskite QDs are entirely coated by PbBr(OH). Therefore, the color of the sample gradually changes to greenish in ambient light, and ultrastable MAPbBr_3_@PbBr(OH) is formed. The energy level of the MAPbBr_3_ QDs and PbBr(OH) was studied by ultraviolet photoelectron spectroscopy (UPS), which can provide both the ionization potential and the valence band (VB) level of the sample, as well as its work function (φ). By combining UPS (Supplementary Fig. 16) and absorption spectra, we can establish the energy level. In Fig. 4h, the CB of the matrix (PbBr(OH)) is higher than that of the QDs, while the VB of the matrix is lower than that of the QDs. As a result, the surface defects are passivated by PbBr(OH), and both electrons and holes are confined in the QDs. The band alignment between the inner QDs and the outer matrix can guarantee exciton generation and high-rate radiative recombination of the QDs, thus resulting in a sharp PL QY.

### Application to LEDs

**Fig. 5:**
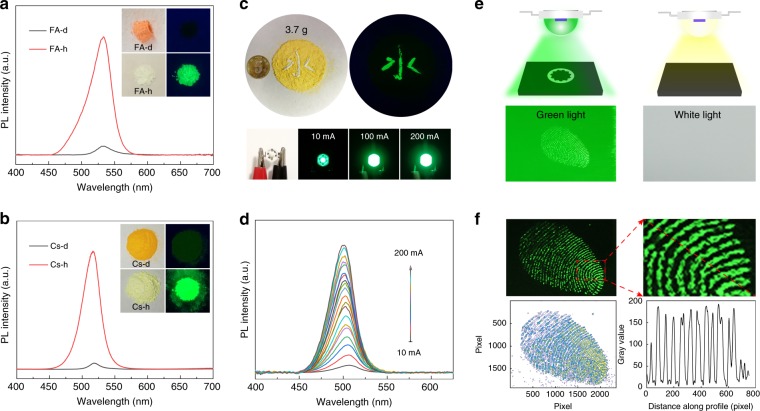
PL spectra of **a** FA lead bromide perovskite and **b** all-inorganic halide perovskites, and the insets show digital images of the corresponding samples in ambient light and under UV light. **c** Digital images of the large-scale synthesized sample (upper images) and LED based on the sample under different driven currents (bottom images). **d** EL spectra of the LEDs under different driven currents from 10 mA to 200 mA. **e** Schematic diagram of potential fingerprint detection based on the as-prepared LED. **f** Photographs of fingerprint (upper images) and the corresponding pseudocolor map and gray values along the profile (bottom images)

Interestingly, this strategy is universal to other bromide perovskites, including FAPbBr_3_ perovskites and CsPbBr_3_. The PL spectra of FAPbBr_3_ are shown in Fig. 5a. The corresponding optical properties, structure, and morphology are shown in Supplementary Figs. 17 and 18. Figure 5b shows the PL spectra of CsPbBr_3_, and the corresponding optical properties, structure, and morphology are shown in Supplementary Figs. 19 and 20. In addition, MAPb(Br/I)_3_@Pb(Br/I)(OH) was prepared by this method, and the corresponding spectrum and image are shown in Supplementary Fig. 21. The fluorescence peak redshifts to ~630 nm, indicating that the emissions can be tuned by changing the X site anion. Notably, this method is simple and facile, and the samples can be prepared on a large scale by proportionally enlarging the precursors, as shown in Fig. 5c. Three grams of the powders are obtained in one synthesis process, and no obvious differences are observed from batch to batch. A Chinese character was written in the samples using water as ink; the regions with water are greenish in color and show bright green fluorescence under UV illumination. As discussed above, compared to traditional perovskites, synthetic powders have improved ambient, thermal, and photostabilities, and the reported perovskites with improved stability are summarized in Table 1. High PL QY and stability can be achieved simultaneously for the first time in this work. Based on these merits, this material can be employed as a promising phosphor in LEDs. UV-pumped LEDs were fabricated by coating the MA-h and PDMS mixture onto 365 nm UV chips. Bright green light with color coordinates of (0.21, 0.52) can be observed (Supplementary Fig. 22), and the emission intensity of the LEDs increases as the drive current increases (Supplementary Fig. 23). The light output of the LED versus time was measured, as shown in Supplementary Fig. 24. The initial luminous intensity of the LED is 1061 cd/m^2^, and the intensity decreases with running time. The luminous intensity of the LED settles at ~400 cd/m^2^ after 480 min (8 h) of running. The luminescence intensity of the as-prepared LEDs can reach 9549 cd/m^2^ when the driving current increases to 200 mA. The corresponding EL spectra of the LEDs under different drive currents from 10 mA to 200 mA are shown in Fig. 5d. In addition, an LED with bright green light can improve the contrast of illuminated objects due to the sensitivity of human eyes to green light. Thus, the as-prepared LEDs are suitable for manual defect detection. As illustrated in Fig. 5e, the light of the LED shines on the surface of the object; thus, the blemishes on the surface of the object (such as scratches, dust, and fingerprints) can be clearly observed. The different defects on the surface of substrates under illumination with a white LED and the as-prepared LED are shown in Supplementary Figs. 25 and 26. Under irradiation with green light, the fingerprint on the glass is extracted (Fig. 5f), and the fingerprint can be observed clearly. The corresponding pseudocolour map and gray values along the lines in Fig. 5f (bottom images) further indicates the resolution of the image.

**Table 1:** Summary of PL properties and stability of perovskite QDs

Strategy	Perovskite	Emission peak (nm)	FWHM (nm)	PL QYs (%)	Stability	Ref.
Compositional engineering	CsPbBr_3_ QDs	513	20	95	90% (30 d, air)	^31^
	FAPbBr_3_ NCs	530	22	85	38% (100 °C)	^51^
	CsPbBr_3_: Mn QDs	514–517	20	90	60% (120 d, air)	^52^
Surface engineering	MAPbBr_3_–APTES NCs	505	42	55	70% (2.5 h, isopropanol)	^53^
	CsPbBr_3_–TDPA QDs	522	22	68	80% (300 min, water)	^54^
	CsPbBr_3_–CTAB QDs	515	39	71	63% (80 min, UV)	^55^
Matrix encapsulation	CsPbBr_3_–Meso-SiO_2_ QDs	515	20	55	60% (100 °C)	^33^
	CsPbBr_3_–Ami-SiO_2_ powders	520	26	56	80% (108 h, UV)	^47^
	CsPbBr_3_–PMMA powders	510	25	45	75% (3 d, air)	^56^
	MAPbBr_3_ powders	518	50	11.7	82% (6 months, water)	^42^
	CsPbBr_3_ powders	508	45	53.9	74% (6 months, water)	^42^
	MAPbBr_3_ powders	514	28	71.5	90% (1 year, water, DMF), 80% (100 °C, water), 50% (60 h, UV)	Our work

In summary, we synthesized bromide-based perovskites whose PL QY can be increased from 2.5% to 71.54% by introducing water and decreases minimally in aqueous solution in 1 year. In addition, the as-synthesized MAPbBr_3_@PbBr(OH) can maintain their luminescence in many kinds of solvents and also exhibit excellent ambient, thermal, and photostabilities. The enhanced stability and PL QY can be attributed to the water-induced MAPbBr_3_@PbBr(OH). PbBr(OH) passivated the defects of the MAPbBr_3_ QDs and confined carriers within the QDs so that MAPbBr_3_@PbBr(OH) could reach high emission efficiency; additionally, PbBr(OH) can prevent the exposure of the QDs to air and moisture, thus increasing the stability. This strategy is universal to MAPbBr_3_, FAPbBr_3_, and CsPbBr_3_. UV chip-pumped LEDs were fabricated by using the sample as promising phosphors, and the luminance intensity of the device was as high as 9549 cd/m^2^. Furthermore, blemishes including scratches, dust, and fingerprints on the surface of glass could be observed under the illumination of the LEDs, indicating that they are suitable for manual defect detection. This efficient approach for the synthesis of ultrastable and highly efficient luminescent perovskites will push forward their practical applications.

## Materials and methods

The materials used were methylamine (CH_3_NH_2_; 32% wt/wt aq. soln), hydrobromic acid (HBr, 48% wt/wt aq. soln), lead bromide (PbBr_2_; >98%, Aladdin), ammonium hydroxide (NH_3_·H_2_O; 27% wt/wt aq. soln), and DMF (>99.8%, Aladdin). All the reagents were used directly without further purification.

### Synthesis of methylammonium bromide

Methylammonium bromide (CH_3_NH_3_Br) was prepared by slowly mixing methylamine with HBr at a 1:1 molar ratio under continuous stirring for 2 h at 0 °C. CH_3_NH_3_Br was then crystallized by removing the solvent from an evaporator. Then, the CH_3_NH_3_Br was washed with diethyl ether three times. White powders were obtained by recrystallization with ethanol. Subsequently, the powders were dried in vacuum for 24 h and stored in a dark and dry environment for further use.

### Synthesis of MAPbBr_3_ perovskites

The pH of DMF was preadjusted with ammonium hydroxide, and the pH was ~9. Then, 2.31 g PbBr_2_ and 0.67 g MABr (the molar ratio of PbBr_2_ to MABr was 1.05) were dissolved in 6 ml alkaline DMF mixed solution to form a white viscous solution, and then the solution was continuously stirred until a white-colored precipitate was formed. Subsequently, the precipitate was centrifuged at 7500 rpm, and the precipitate was placed into an oven at 70 °C to obtain MAPbBr_3_ perovskite powders. MAPbBr_3_ can be converted to bright emissive MAPbBr_3_@PbBr(OH) through the addition of water.

### Synthesis of MAPbBr_3_@PbBr(OH)

The synthesis details of MAPbBr_3_@PbBr(OH) are as follows: 1 g of the as-prepared MAPbBr_3_ powder was added to 10 ml of water, and then the mixture was stirred for 1 min. The process was replicated several times until the yellow MAPbBr3 powder transformed into greenish MAPbBr_3_@PbBr(OH) powder.

### Synthesis of MAPb(Br/I)_3_@Pb(Br/I)(OH)

One gram of the as-prepared MAPbBr_3_@PbBr(OH) was homogeneously mixed with KI at a mass ratio of 1:2, and then MAPb(Br/I)_3_@Pb(Br/I)(OH) was obtained after several minutes.

### Fabrication of LEDs

First, the as-prepared MA-h phosphors were uniformly mixed with PDMS. The mixture of MA-h and PDMS was debubbled, and coated onto a 365 nm UV chip and then cured in an oven at 70 °C for 1 h to obtain UV-pumped LEDs.

### Characterization

The XRD patterns were obtained by an X’Pert Pro diffractometer. The TEM images were taken by a transmission electron microscope (JEM-2010), and the SEM images were obtained on a JEOL JSM6700F field-emission scanning electron microscope. The Fourier transform infrared (FTIR) spectra of the samples were recorded on a Thermo Scientific Nicolet iS10 FTIR spectrometer. The PL QYs of the samples were measured by an Edinburgh fluorescence spectrometer (FLS980). The X-ray photoelectron spectra of the samples were collected by a Thermo Fisher Scientific ESCALAB 250Xi spectrometer equipped with an Al Ka X-ray radiation source, and the XPS binding energy was internally referenced to the C 1 s peak (BE = 284.8 eV). The PL spectra were measured by a Hitachi F-7000 spectrophotometer, and the UV–vis absorption spectra were characterized by a UH4150 spectrophotometer.

### Computational methods

All the first-principle calculations were performed utilizing the plane-wave pseudopotential approach within density functional theory as implemented in the Vienna Ab initio Simulation Package. The 1 s for H, 2s^2^2p^2^ for C, 2s^2^2p^3^ for N, 2s^2^2p^4^ for O, 6s^2^6p^2^ for Pb, and 4s^2^4p^5^ for Br were treated as valence electrons, and the interactions between nucleus and valence electrons were described by the projected augmented wave method. The electronic wave functions were expanded in plane-wave basis sets with a kinetic energy of 400 eV, and Brillouin zone sample meshes with 2π × 0.02 Å^‒1^ were utilized to ensure the energy convergence of our calculations.

## Supplementary information

Supplementary Information for Water-Induced MAPbBr3@PbBr(OH) with Enhanced Luminescence and StabilitySupplementary Information for

## Data Availability

The data that support the findings of this study are available from the corresponding authors upon reasonable request.
