# Supplementary material for: Water-induced MAPbBr3@PbBr(OH) with enhanced luminescence and stability
Source: Light Sci Appl. 2020 Mar 17;9:44. doi: 10.1038/s41377-020-0283-2 (PMC7078192; doi:10.1038/s41377-020-0283-2)
Supplement: Supplementary file 1 — Supplementary Information for Water-Induced MAPbBr3@PbBr(OH) with Enhanced Luminescence and StabilitySupplementary Information for [file 41377_2020_283_MOESM1_ESM.docx]

Supplementary Information for

Water-Induced MAPbBr_3_@PbBr(OH) with Enhanced Luminescence and Stability

*Kai-Kai Liu^1^,Qian Liu^1^, Dong-Wen Yang^1^, Ya-Chuan Liang^1^, Lai-Zhi Sui^2^, Jian-Yong Wei^1^, Guo-Wei Xue^1^, Wen-Bo Zhao^1^, Xue-Ying Wu^1^, Lin Dong^1*^, Chong-Xin Shan^1*^*

^1^ Henan Key Laboratory of Diamond Optoelectronic Materials and Devices, Key Laboratory of Material Physics, Ministry of Education, School of Physics and Microelectronics, Zhengzhou University, Zhengzhou 450052, China

^2^ State Key Laboratory of Molecular Reaction Dynamics, Dalian Institute of Chemical Physics, Chinese Academy of Sciences, 457 Zhongshan Road, Dalian, 116023, China.

* To whom the correspondence should be addressed. E-mail: [ldong@zzu.edu.cn](mailto:ldong@zzu.edu.cn), E-mail: [cxshan@zzu.edu.cn](mailto:cxshan@zzu.edu.cn).


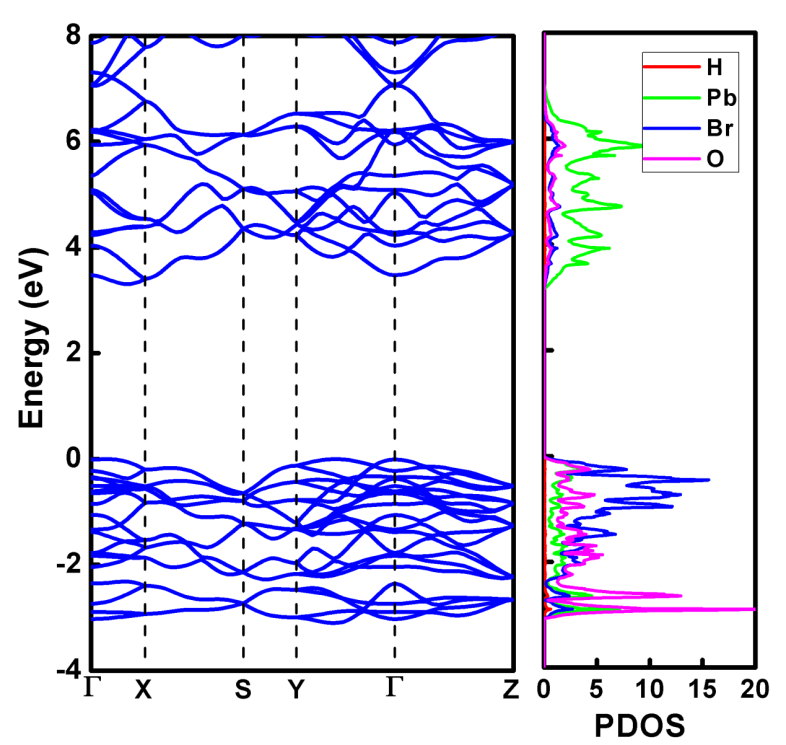


Figure S1. The band structure and density of states of PbBr(OH) compound.


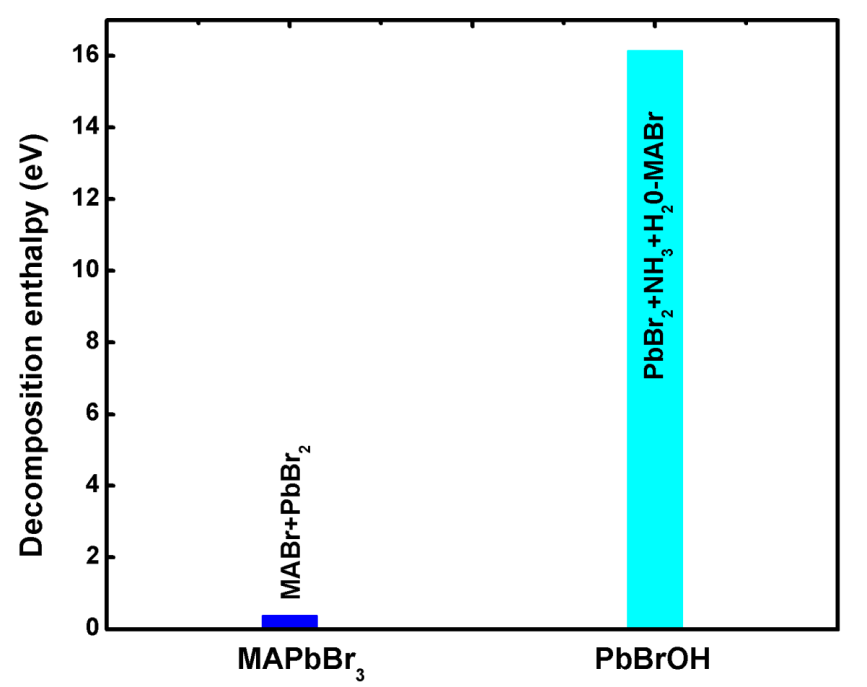


Figure S2. The decomposition enthalpies corresponding to their decomposition pathways for MAPbBr_3_ and PbBr(OH), respectively.

Table S1. The decomposition enthalpies and band gaps of MAPbBr_3_ and PbBr(OH), respectively.

|  | Decomposition enthalpy (eV/f.u.) | Band gap (eV) |
| --- | --- | --- |
| MAPbBr_3_ | 0.38 | 1.97 |
| PbBrOH | 16.15 | 3.1 |


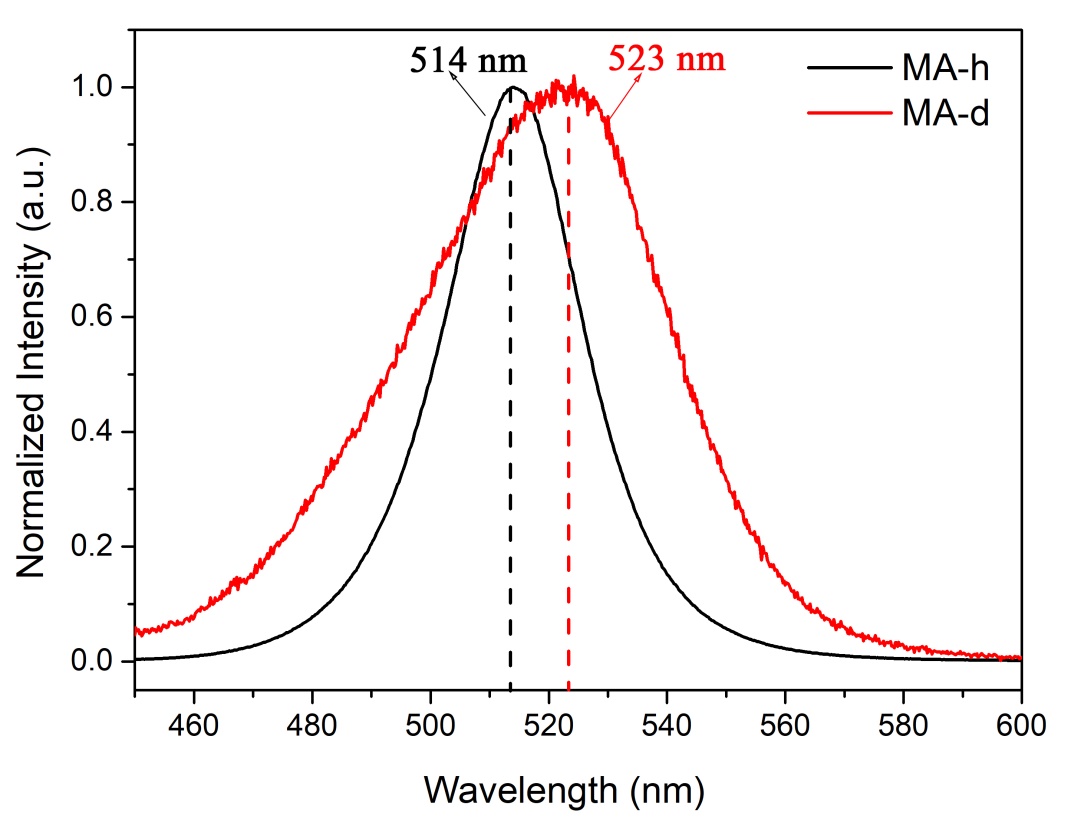


Figure S3. Normalized PL spectra of MA-h and MA-d.


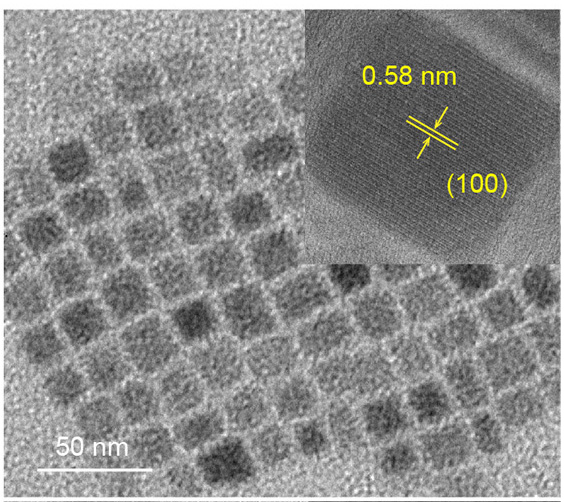


Figure S4. TEM image of the crushed MA-d, inset is the HRTEM image.


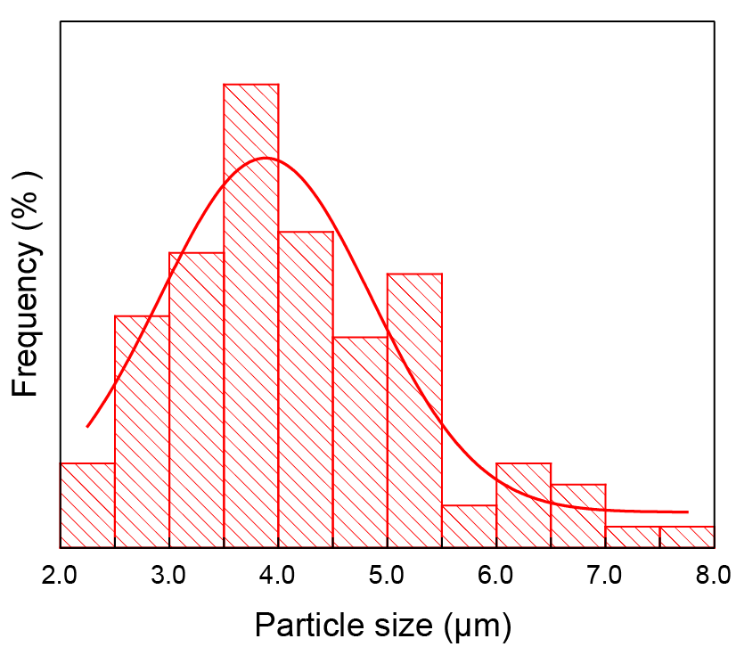


Figure S5. Histogram for the size distribution of MA-h.


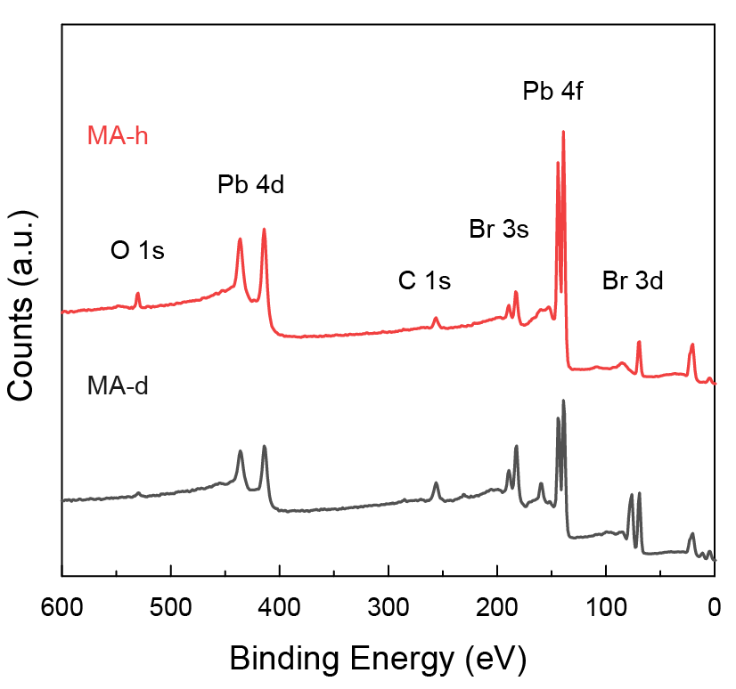


Figure S6. XPS survey spectra of MA-d and MA-h.

| Sample | Br (At%) | Pb (At%) | Br/Pb |
| --- | --- | --- | --- |
| MA-d | 56.84 | 18.56 | 3.06 |
| MA-h | 36.7 | 33.02 | 1.11 |

Table S2. Atomic ratios of Pb and Br elements of the MA-d and MA-h characterized by XPS.


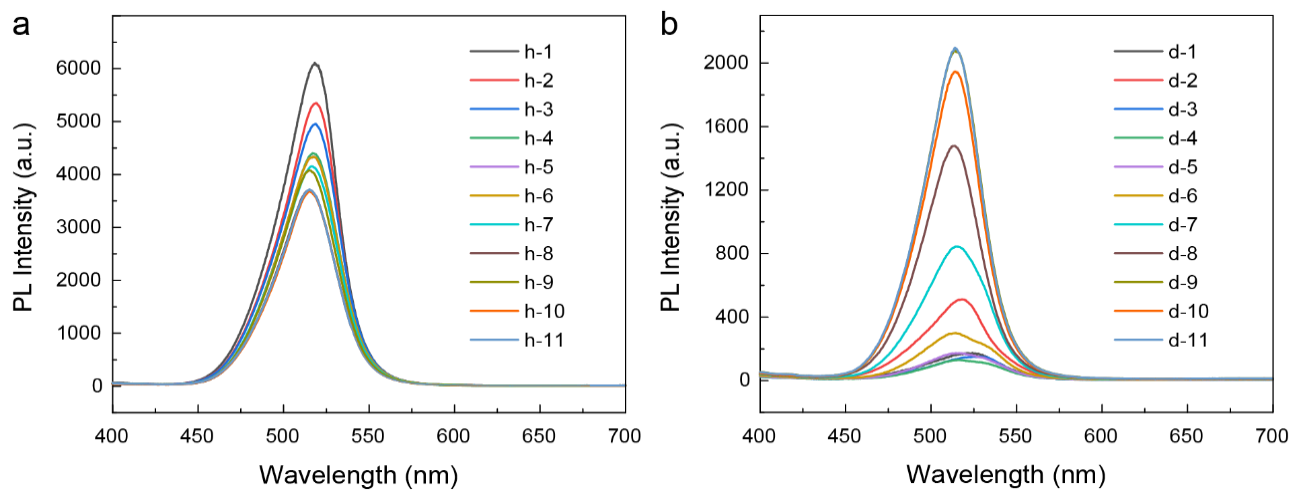


Figure S7. PL spectra of a) MA-h and b) MA-d at hydrate-dehydrate cycle test.


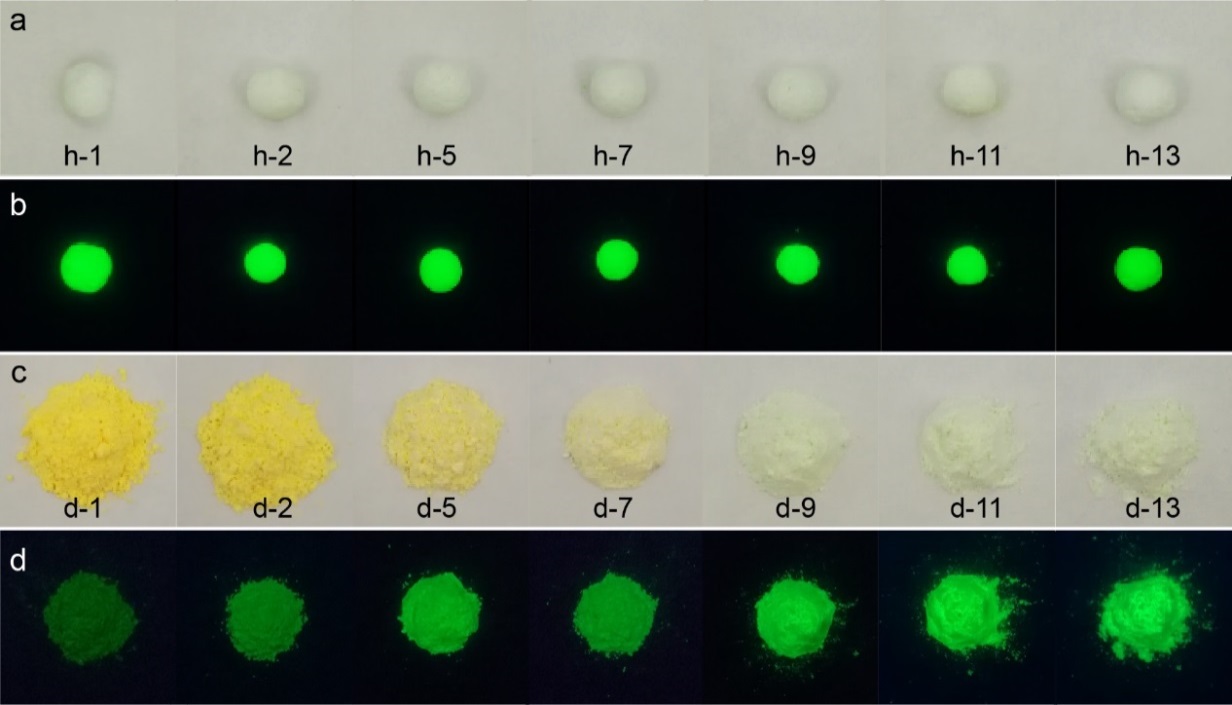
Figure S8. The photographs of MA-h and MA-d at hydrate-dehydrate cycle test. The photographs of MA-h a) in ambient light and b) under UV light. The photographs of MA-d c) in ambient light and d) under UV light.


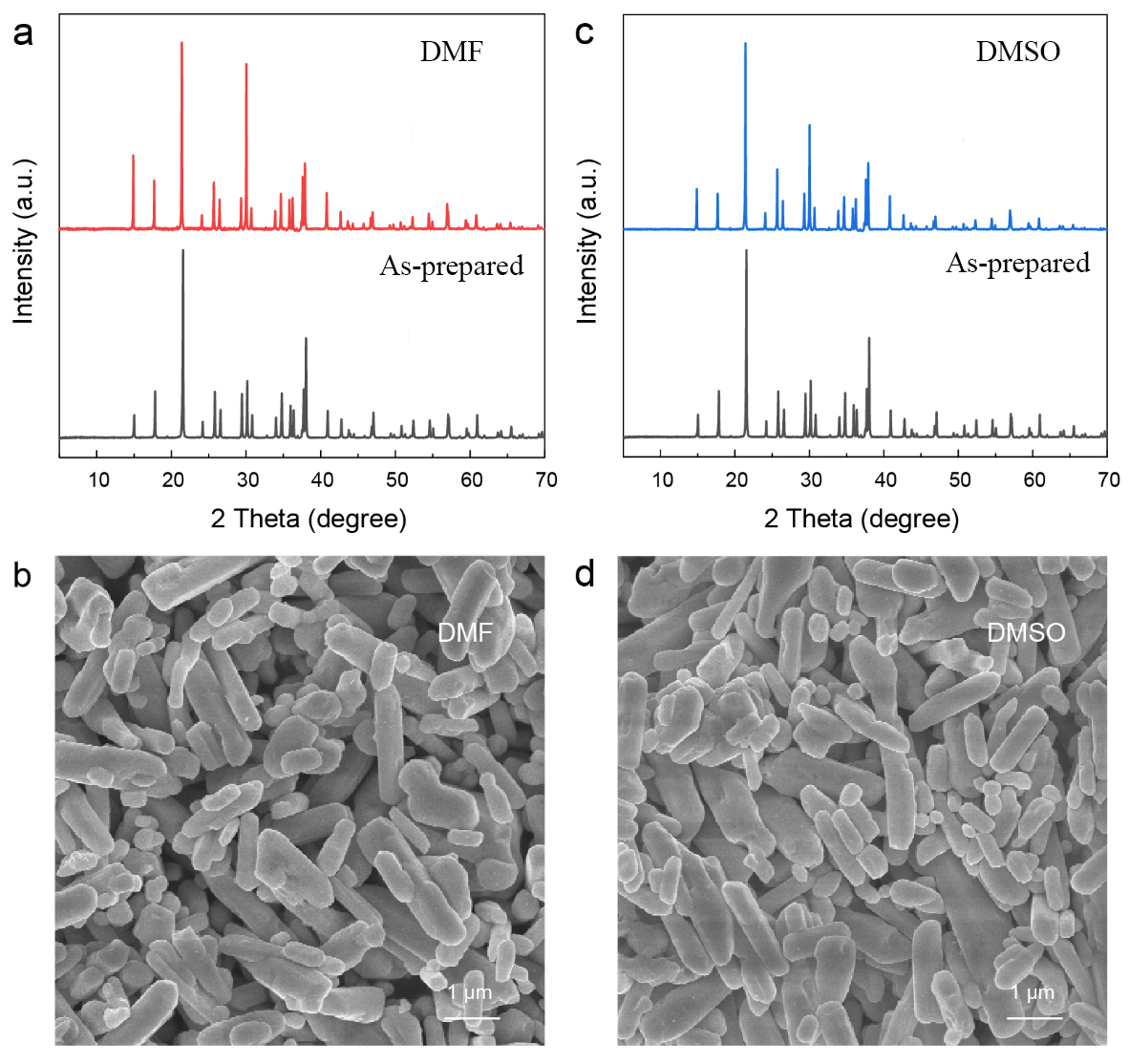


Figure S9. XRD patterns and SEM images of MA-h immersed in DMF and DMSO.


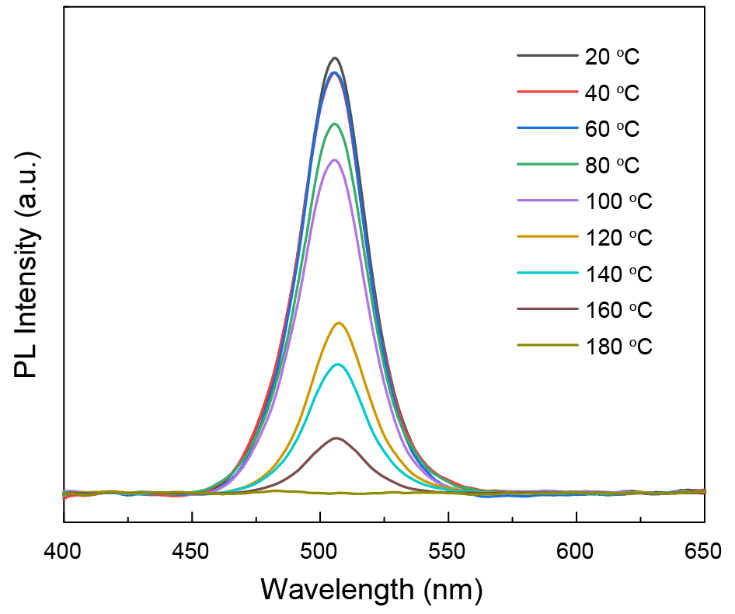


Figure S10. PL spectra of MA-h treated at different temperatures.


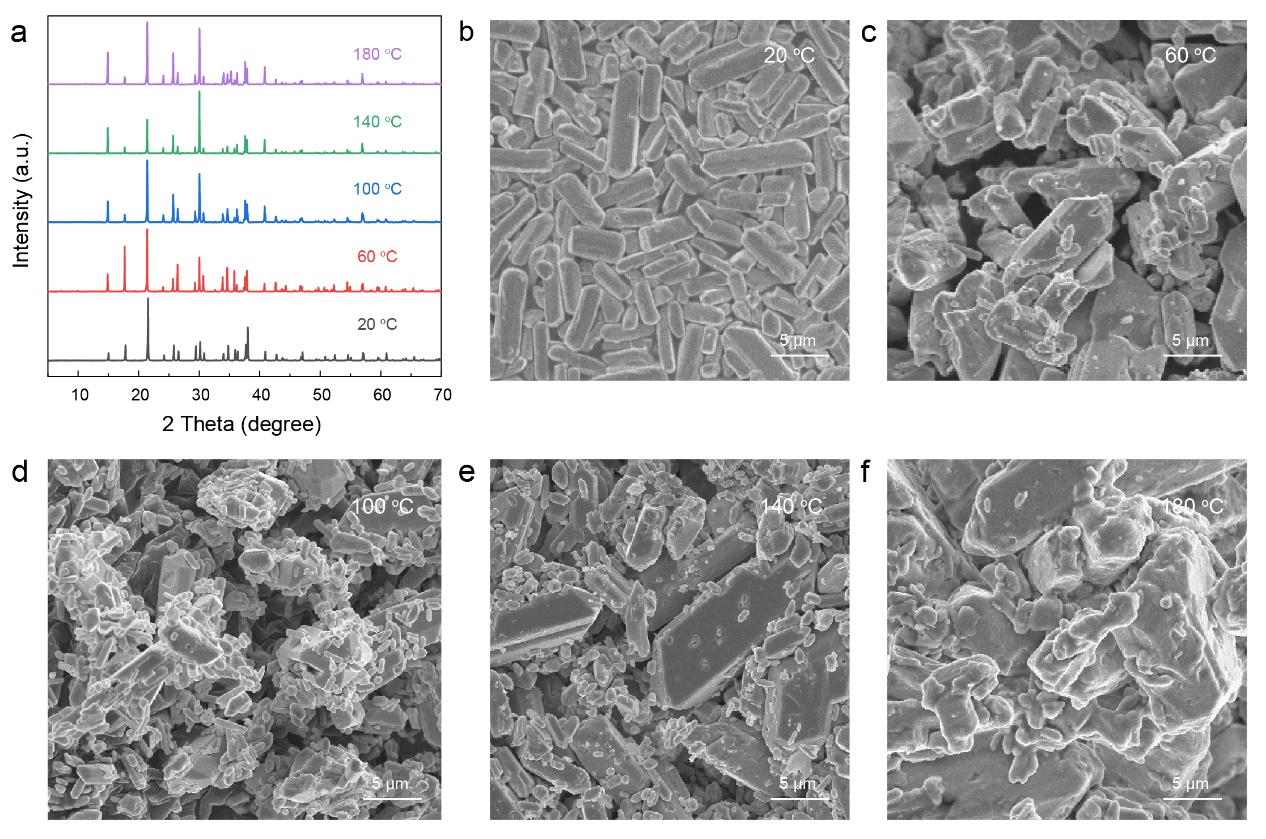


Figure S11. XRD patterns and SEM images of MA-h treated at different temperatures.


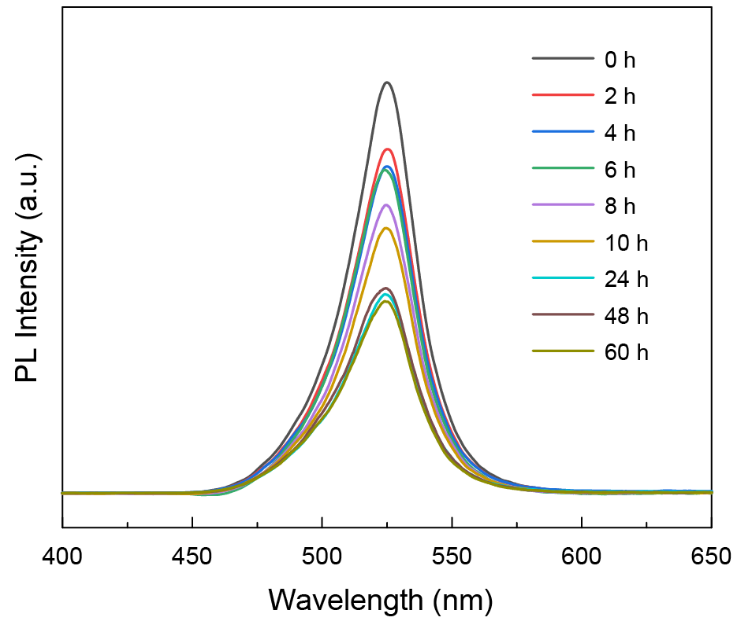


Figure S12. PL spectra of MA-h under irradiation of UV light (365 nm) continuously.


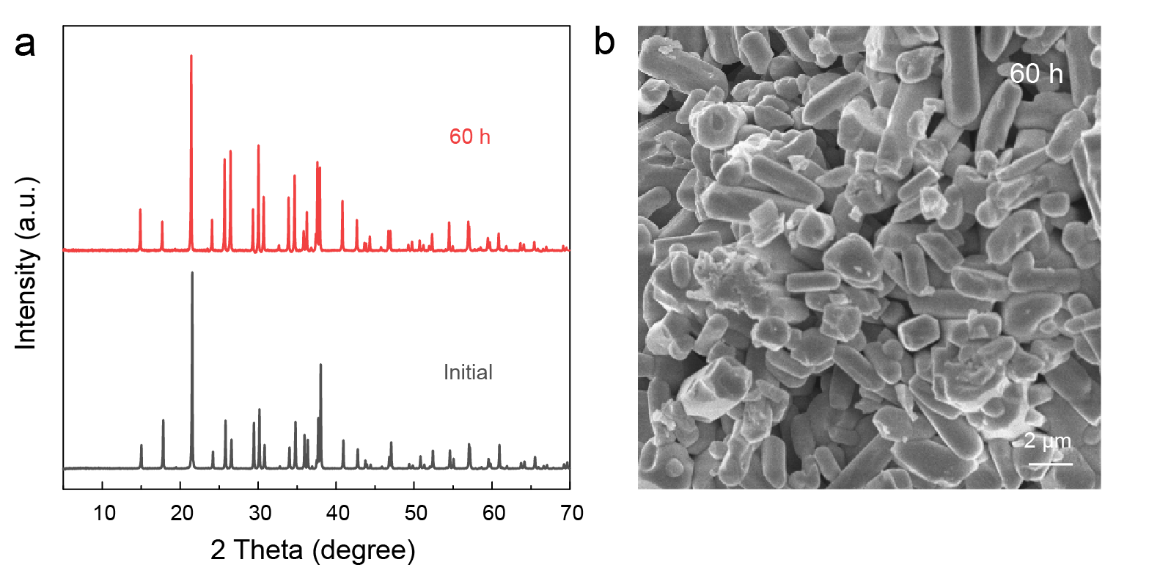


Figure S13. XRD patterns and SEM image of the MA-h after 60 h UV irradiation continuously.

| Sample | R^2^ | A_1_ | τ_1_  (ns) | A_2_ | τ_2_  (ns) | A_3_ | τ_3_  (ns) | τ_ave_  (ns) |
| --- | --- | --- | --- | --- | --- | --- | --- | --- |
| MA-d | 0.99 | 6.52E+17 | 1.08 | 1.75E+04 | 10.37 | 1.41E+03 | 108.94 | 1.08 |
| MA-h | 0.99 | 6.29E+09 | 2.50 | 4702.331 | 22.58 | 950.7462 | 273.11 | 2.50 |

Table S3. The fitted lifetimes of the samples


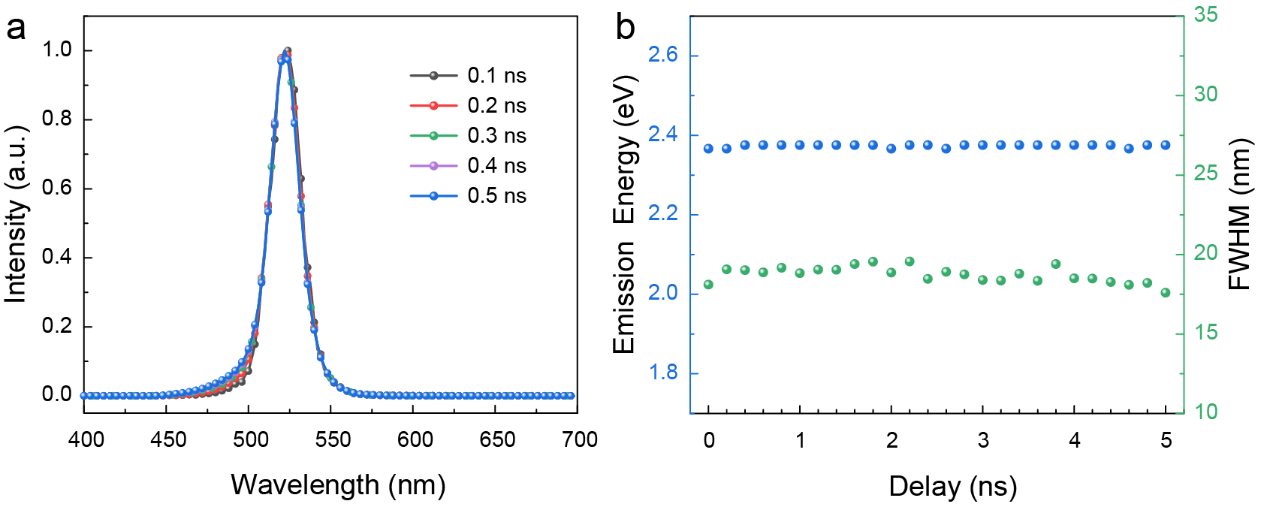


Figure S14. a) Normalized PL spectra and b) FWHM and emission peak of MA-h at different times.


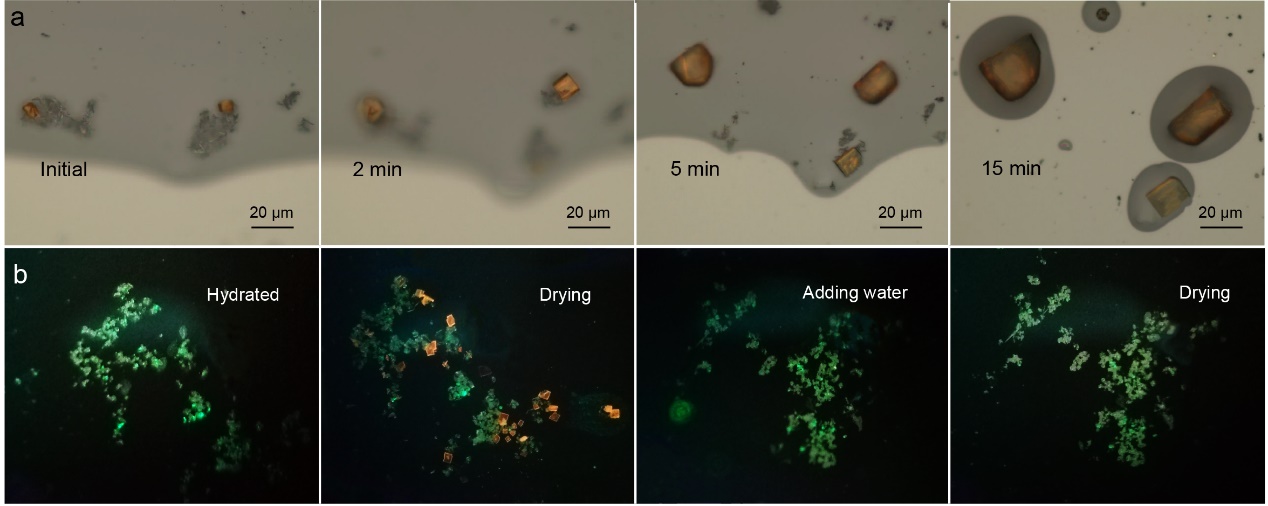
Figure S15. Morphological evolution of MA-h during drying process. a) Optical microscopy images. b) Fluorescence microscopy images.


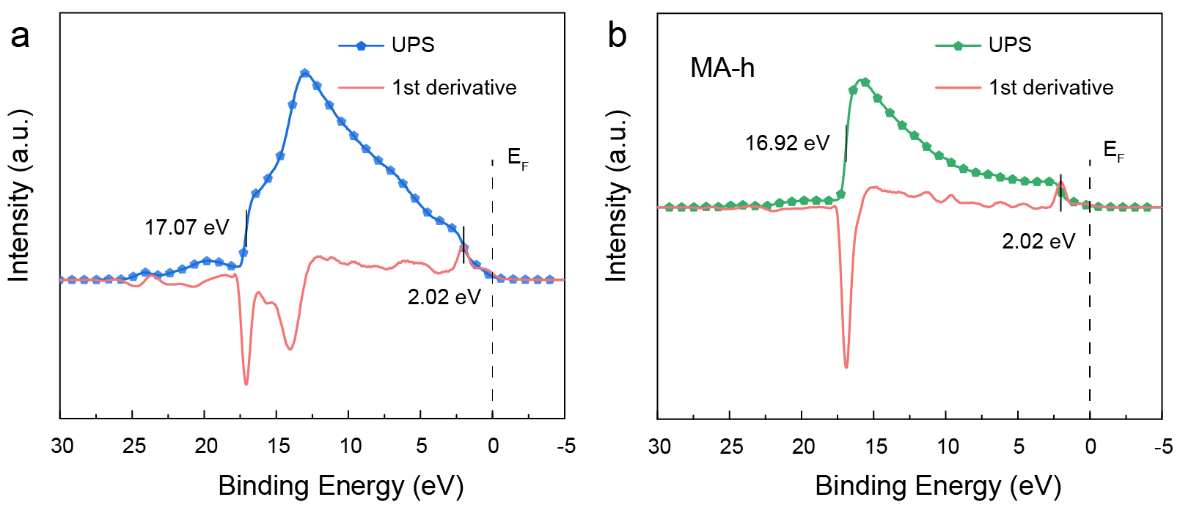


Figure S16. UPS spectra of a) MA-d and b) MA-h.


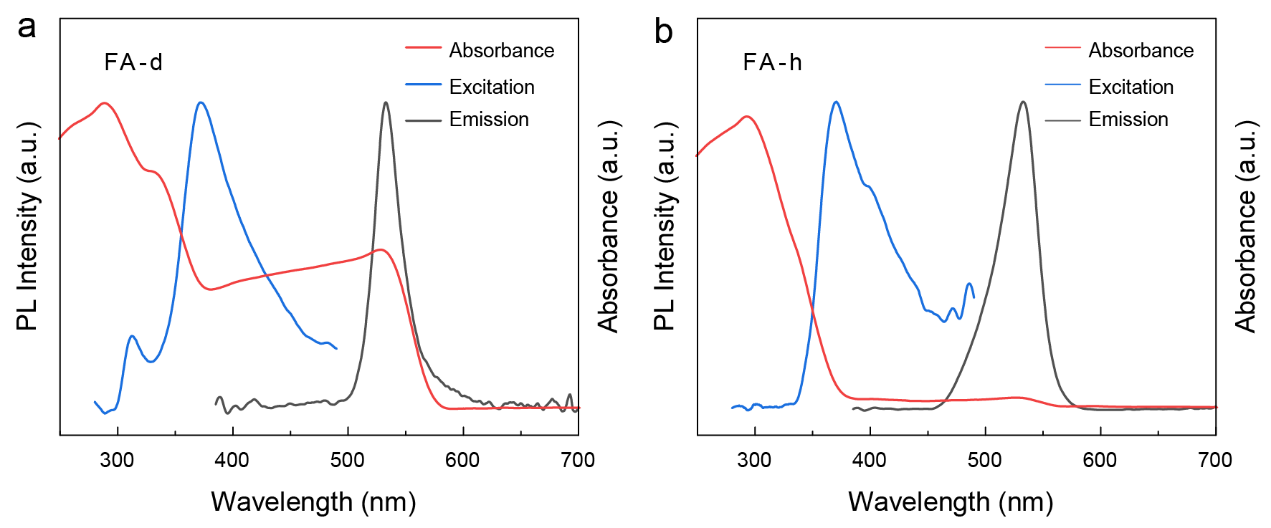


Figure S17. PL, PLE and absorption spectra of FAPbBr_3_ a) before (FA-d) and b) after (FA-h) addition of water.


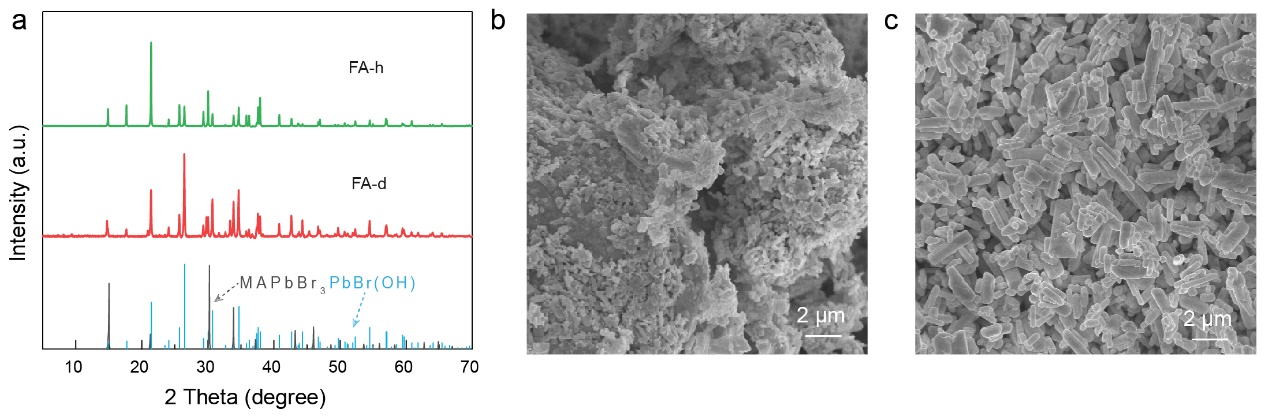


Figure S18. a) XRD patterns of FA-d and FA-h. The SEM images of b) FA-d and c) FA-h.


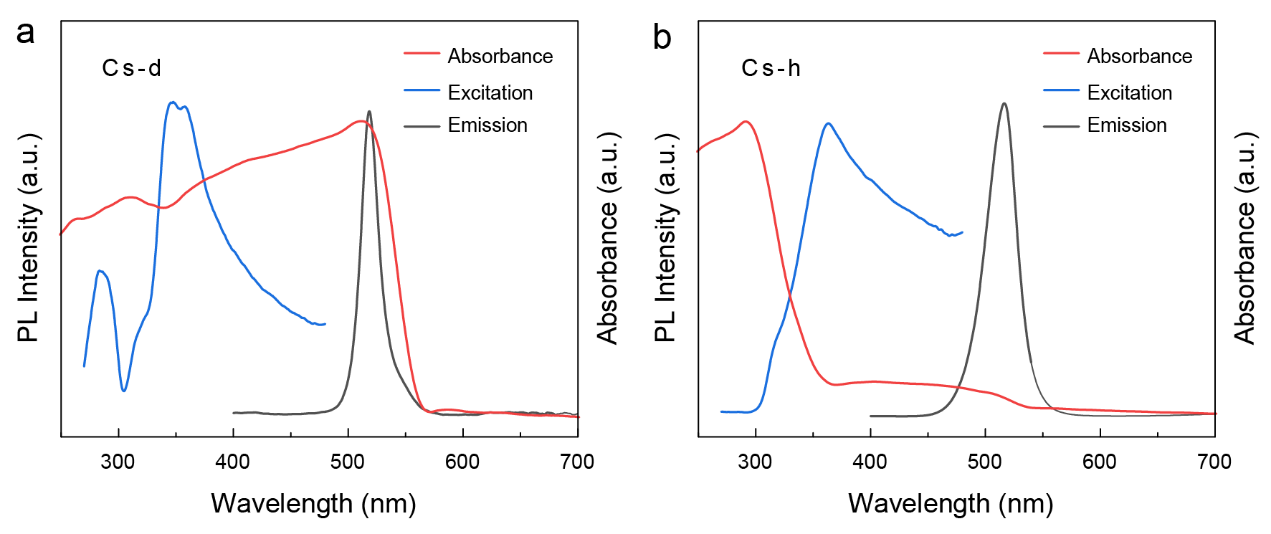
Figure S19. PL, PLE and absorption spectra of CsPbBr_3_ a) before (Cs-d) and b) after (Cs-h) addition of water.


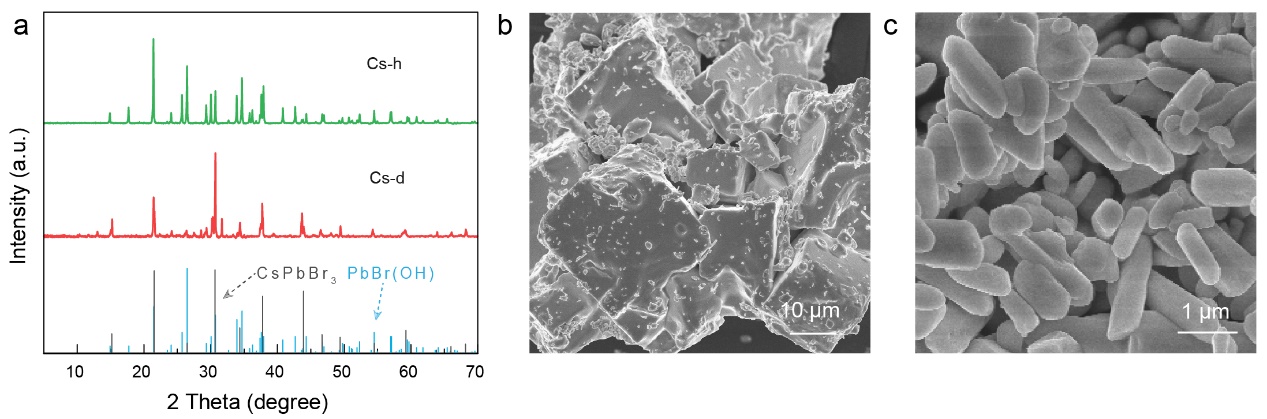


Figure S20. a) XRD patterns of Cs-d and Cs-h. The SEM images of b) Cs-d and c) Cs-h.


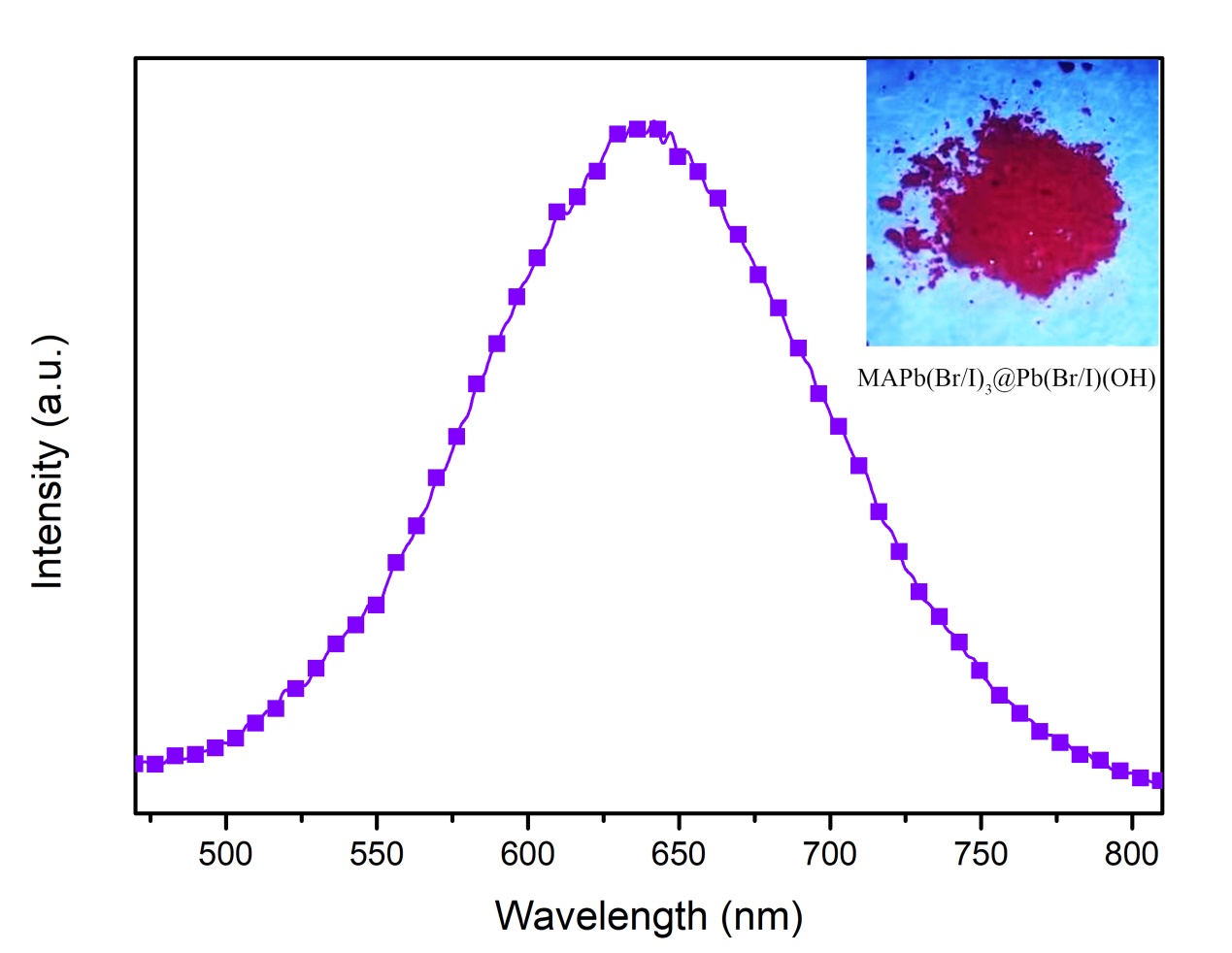


Figure S21. PL spectrum of MAPb(Br/I)_3_@Pb(Br/I)(OH), the inset is the fluorescence image.


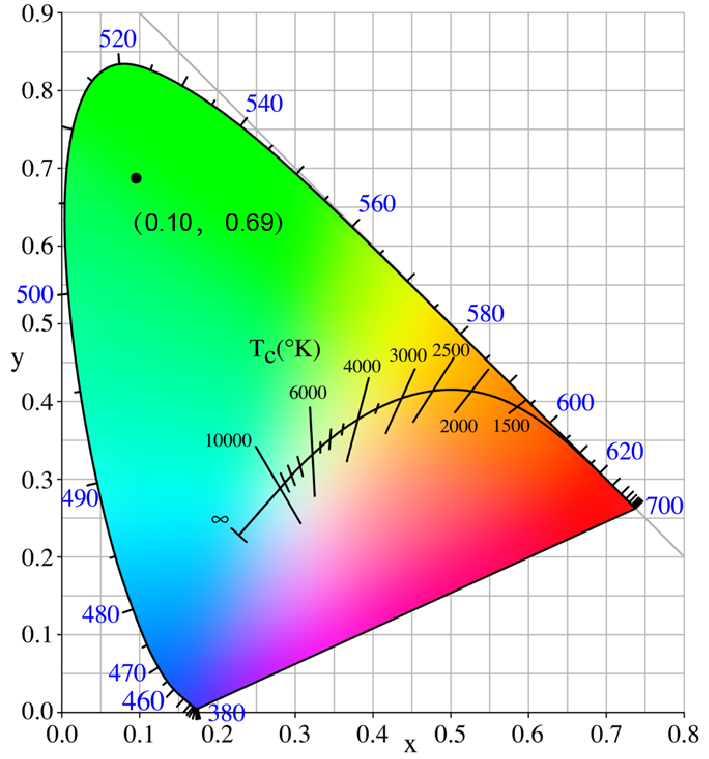


Figure S22. CIE chromaticity coordinate of the as-prepared LED.


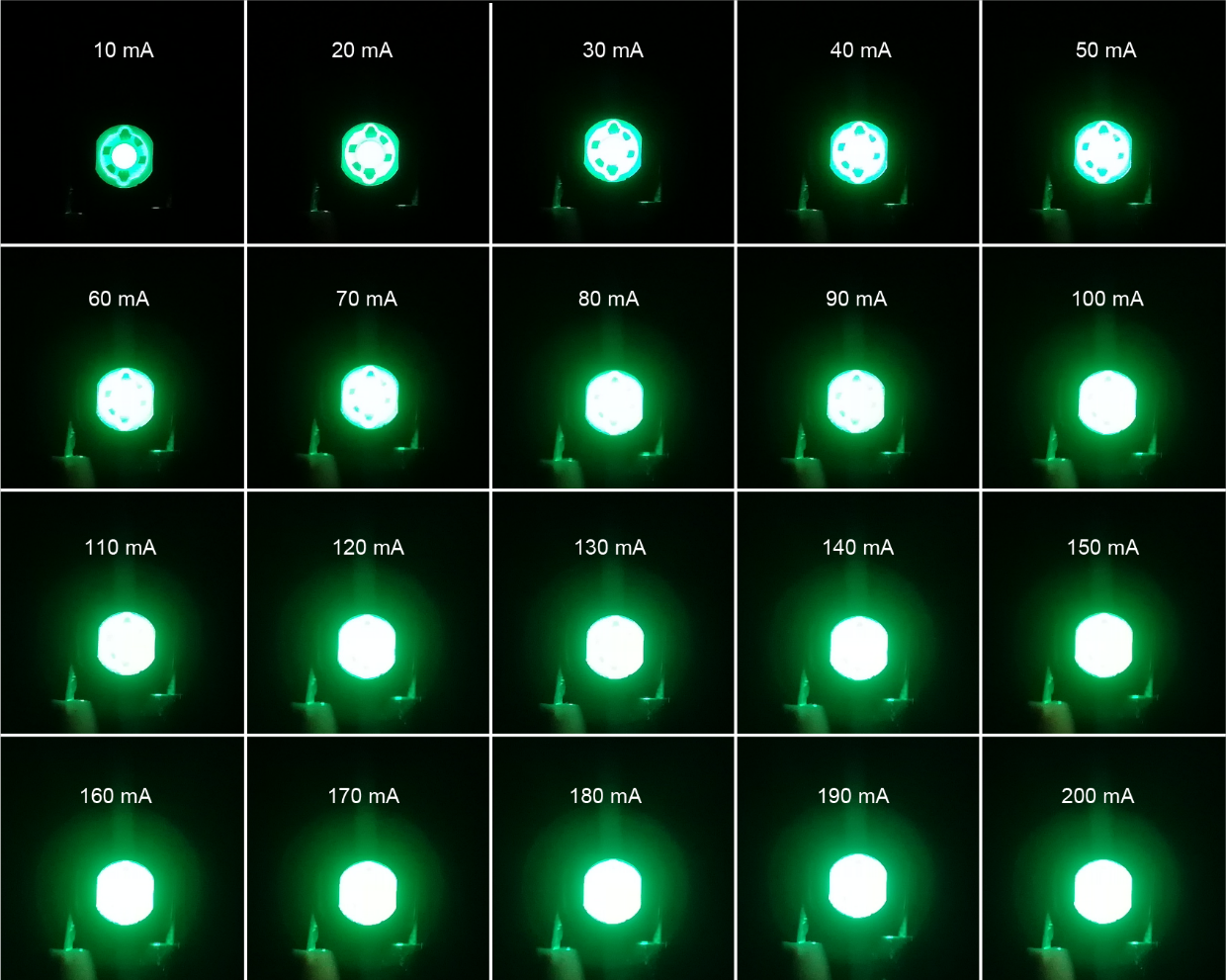
Figure S23. Images of the LED under different driven currents from 10 mA to 200 mA.


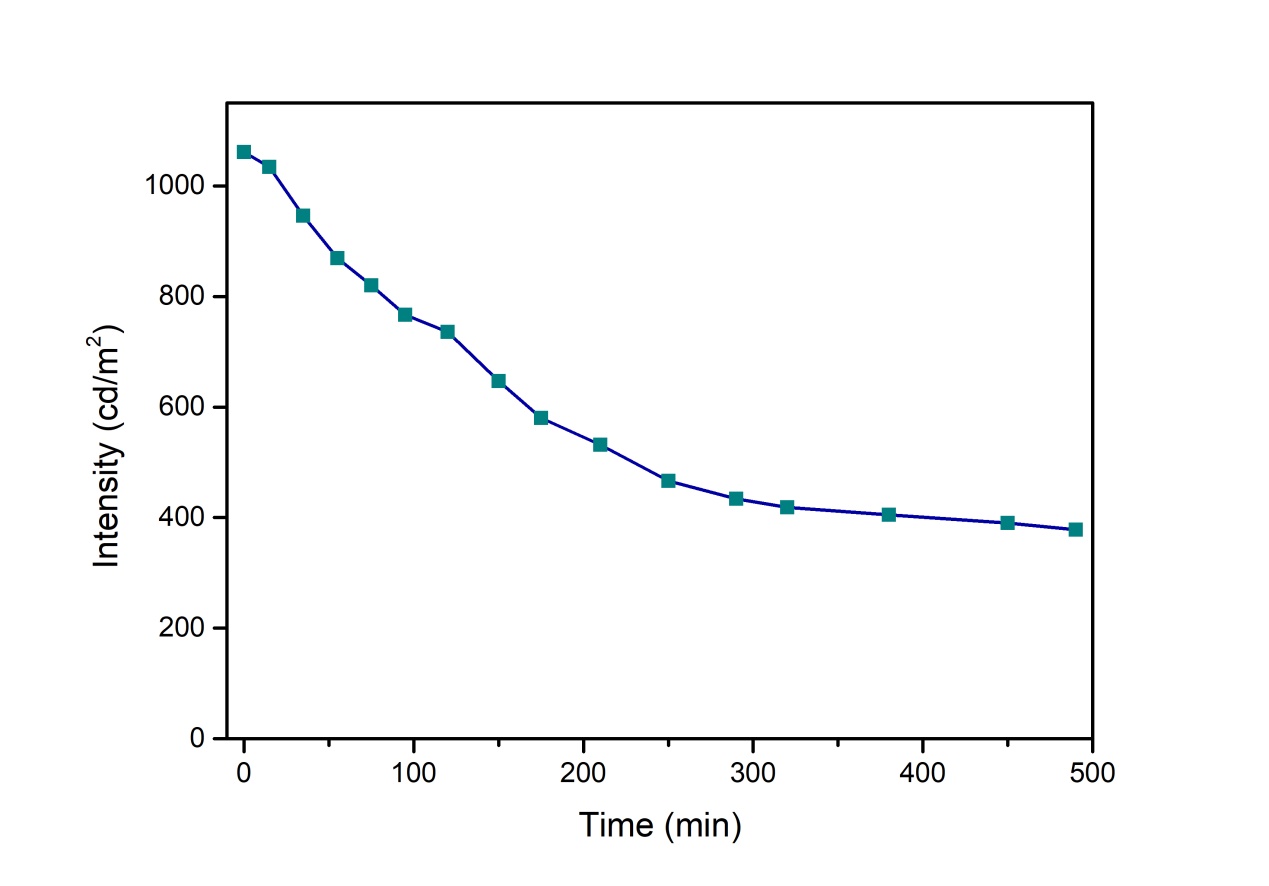


Figure S24. The emission intensity of the LED versus operation time.


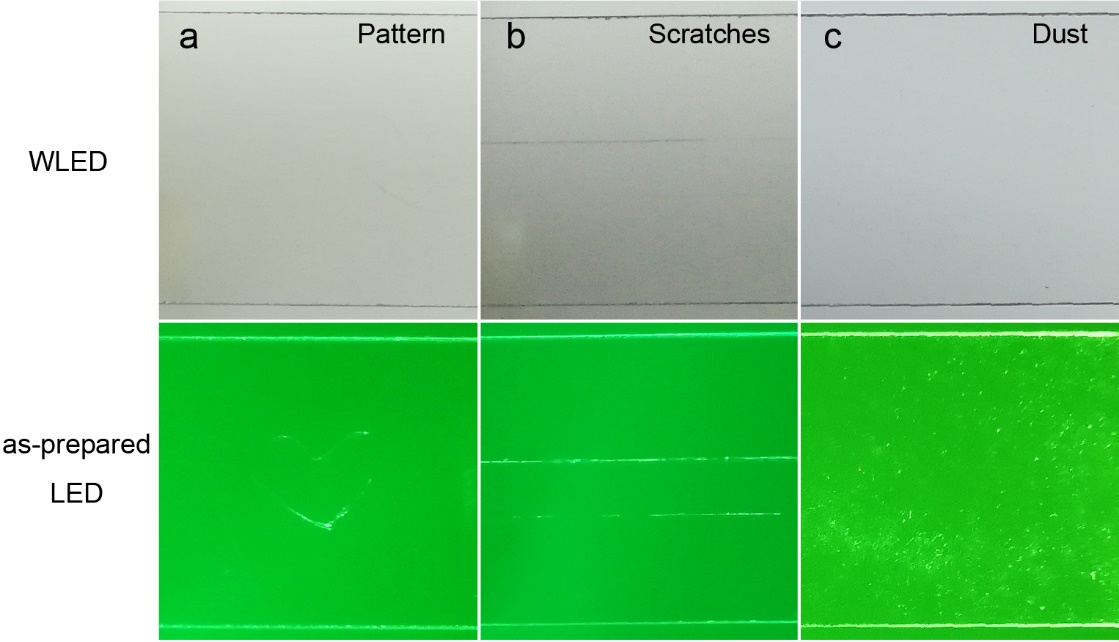


Figure S25. Photographs of a) pattern, b) scratches, and c) dust on the surface of glass under the illumination of WLED (Top) and the as-prepared LED (Bottom).


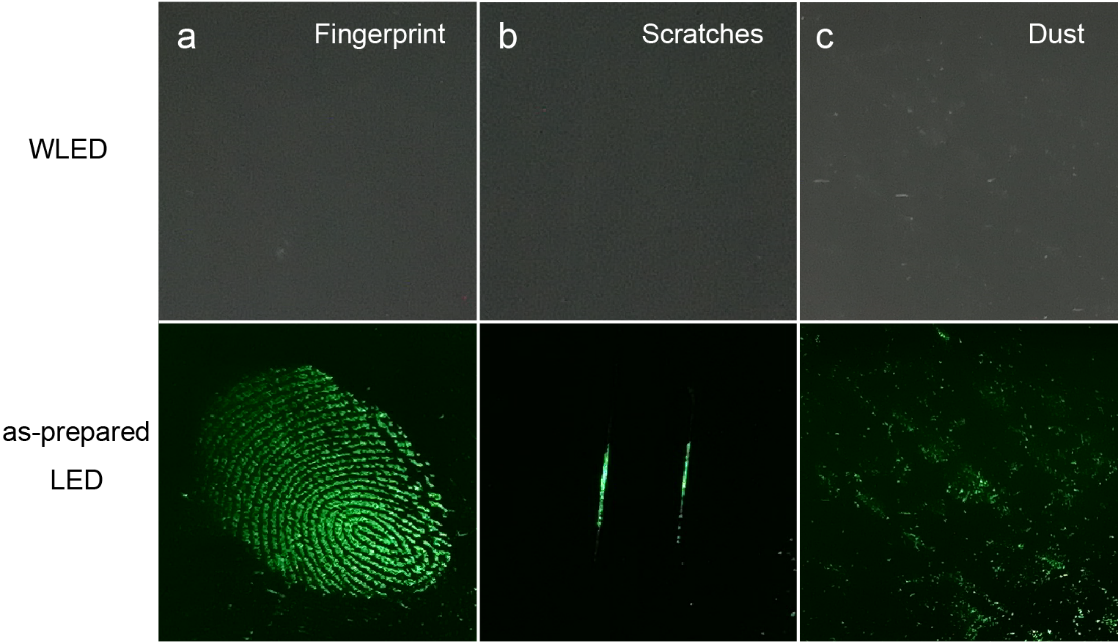


Figure S26. Photographs of a) fingerprint, b) scratches, and c) dust on the surface of Si wafer under the illumination of WLED (Top) and the as-prepared LED (Bottom).
